# Results of Antibiotic-Impregnated Cement/Polymer-Coated Intramedullary Nails in the Management of Infected Nonunion and Open Fractures of Long Bones

**DOI:** 10.7759/cureus.43421

**Published:** 2023-08-13

**Authors:** Tarun Solanki, Maneesh K Maurya, Pankaj K Singh

**Affiliations:** 1 Orthopaedics, KVR Hospital Kashipur, Kashipur, IND; 2 Orthopaedics, Anamika Orthopedic Centre and Multispeciality Hospital, Hajipur, IND

**Keywords:** functional outcome, nail cement, long bones, open fractures, non-union, intramedullary nails

## Abstract

Background

Fractures of long bones can sometimes lead to complications such as infection or nonunion, resulting in significant patient morbidity. Surgical intervention and antibiotics are often necessary to treat these complications. Antibiotic-impregnated cement/polymer-coated intramedullary nails have emerged as an effective surgical treatment for infected nonunion and open fractures. These implants elude high concentrations of antibiotics at the infection site while stabilizing the fracture. Extensive research has shown promising results, with success rates ranging from 60% to 100%. Benefits of these implants include stable fracture fixation, early weight-bearing, and reduced need for prolonged antibiotic therapy. However, concerns remain regarding antibiotic resistance and potential toxicity. This study aims to evaluate the efficacy and safety of these implants in managing infected nonunion and open fractures of the femur and tibia.

Methods

This prospective hospital-based study aimed to assess the efficacy and safety of antibiotic-impregnated cement/polymer-coated intramedullary nails for managing infected nonunion and open fractures of the femur and tibia. The study included patients aged 18 or older who received treatment with these implants between January 1, 2021 and December 31, 2022. Patients allergic to vancomycin or teicoplanin, with gap nonunion >2 cm, or lost to follow-up were excluded. Data on demographics, fracture details, previous treatment, surgery, antibiotics, and outcomes were collected using a structured proforma. Surgeries involved implant removal, debridement, culture testing, reaming, fracture reduction, and stabilization with an antibiotic-impregnated cement/polymer-coated intramedullary nail. Postoperatively, patients received antibiotics, had wound inspections, and were gradually allowed weight-bearing. Follow-up appointments and radiographic/laboratory assessments were conducted at regular intervals. The primary outcome was successful bone union, and secondary outcomes included time to union, infection rate, nonunion rate, and revision surgery.

Results

The majority of participants were male, with a mean age of 39.76 years. Most fractures were Gustilo-Anderson grade 3 (46.7%) and involved the tibia (73.3%). The mean bone gap after debridement was 1.3 cm. The median follow-up period was 8.21 months. Infection was controlled in 93.3% of patients, with the tibia being the most common site (70.0%). Successful bone union was achieved in 90.0% of patients, with a mean union rate of 22.13 weeks for tibial fractures and 17.21 weeks for femoral fractures. Among patients with bone union, 60.0% did not require additional procedures. Most patients had excellent bony (76.7%) and functional (70.0%) outcomes. The most common complications were the persistence of bone nonunion, impingement of proximal nail, and debonding of nail cement, each occurring in 10.0% of patients.

Conclusion

The study concluded that antibiotic-impregnated cement/polymer-coated intramedullary nails are effective in managing infected nonunion and open fractures of the femur and tibia. The procedure demonstrated a high success rate in controlling infections (93.3%) and achieving bone union (90.0%). Paley's criteria showed excellent bony and functional outcomes in the majority of patients. These findings support the use of this treatment option for such fractures.

## Introduction

Fractures of long bones are common orthopedic injuries that usually heal without complications. However, in some cases, these fractures can become complicated by infection or nonunion, leading to significant morbidity for the patient. Treatment of these complications can be challenging and often requires the use of antibiotics and surgical intervention. One of the surgical treatments used in such cases is the use of antibiotic-impregnated cement/polymer-coated intramedullary nails [1,2].

Antibiotic-impregnated cement/polymer-coated intramedullary nails have been shown to be effective in the treatment of infected nonunion and open fractures of long bones. The use of these implants is based on the principle of providing high concentrations of antibiotics at the site of infection while also stabilizing the fracture [3]. The antibiotic-impregnated cement/polymer coating on the intramedullary nail serves as a local drug delivery system, which releases antibiotics into the surrounding tissue over a prolonged period, thereby maintaining high drug concentrations at the site of infection [3].

The use of antibiotic-impregnated cement/polymer-coated intramedullary nails has been extensively studied in the past few decades, and the results have been promising. Several studies have reported a high success rate of using these implants in the management of infected nonunion and open fractures of long bones [4,5]. The success rates reported range from 60% to 100%, depending on the severity of the infection and the type of antibiotic used [5].

One of the advantages of using antibiotic-impregnated cement/polymer-coated intramedullary nails is that they provide stable fixation of the fracture, which is essential for successful healing [6]. The use of these implants also allows for early weight-bearing, which can significantly reduce the time required for rehabilitation. Additionally, the use of antibiotic-impregnated cement/polymer-coated intramedullary nails reduces the need for prolonged antibiotic therapy, which can lead to the development of antibiotic resistance [6].

Despite the promising results reported in the literature, there are still some concerns regarding the use of antibiotic-impregnated cement/polymer-coated intramedullary nails. One of the concerns is the risk of antibiotic resistance, as the prolonged release of antibiotics can promote the development of resistant strains of bacteria. Another concern is the potential for the release of toxic substances from the cement/polymer coating, which can lead to tissue damage and delayed healing [7].

As the use of antibiotic-impregnated cement/polymer-coated intramedullary nails has become an established treatment modality for infected nonunion and open fractures of long bones, the present study was conducted with the aim of evaluating the efficacy and safety of antibiotic-impregnated cement/polymer-coated intramedullary nails in the management of infected nonunion and open fractures of the femur and tibia.

## Materials and methods

Study design

This hospital-based prospective study was designed to evaluate the efficacy and safety of antibiotic-impregnated cement/polymer-coated intramedullary nails in the management of infected nonunion and open fractures of the femur and tibia.

Study population

All patients (18 years or more) with infected nonunion fractures of the femur and tibia who were treated with antibiotic-impregnated cement/polymer-coated intramedullary nails between January 1, 2021 and December 31, 2022 were eligible for inclusion in the study. Patients who were allergic to vancomycin or teicoplanin, with a radiologically visible or intraoperative finding of gap nonunion of >2 cm, or who were lost to follow-up were excluded from the study.

Data collection

Patient data were collected prospectively using a structured proforma which included demographic information, fracture location and classification, previous treatment history, surgical details, antibiotic regimen, and postoperative outcomes. Radiographic and laboratory data were also collected at regular intervals during follow-up visits.

Surgical technique

**Figure 1:**
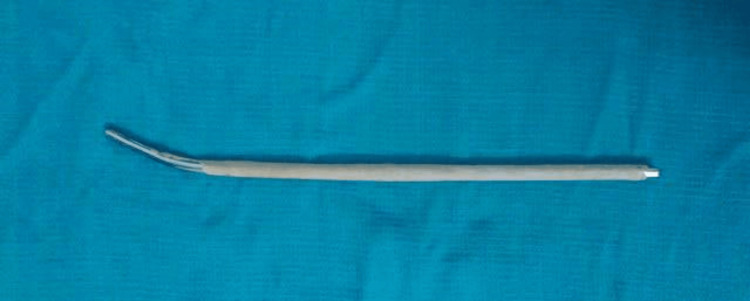
Antibiotic-impregnated cement/polymer-coated intramedullary nail (K-nail). K-nail: Küntscher nail.

All surgeries were performed under general or regional anesthesia. Before surgery, a comprehensive preoperative evaluation was conducted, and informed consent was obtained. After surgical preparation and draping, a longitudinal incision was made over the fracture site. In cases where the patient had undergone previous surgery, the first step involved removing the implant. Next, the infected bone and soft tissues were debrided thoroughly, and copious lavage was performed. Samples of bone, soft tissue, and any purulent material were sent for culture and sensitivity testing. Following this, the intramedullary canal was adequately reamed and prepared to fit a larger diameter nail, which was thoroughly washed with saline. The fracture was then reduced and stabilized using an antibiotic-impregnated cement/polymer-coated intramedullary nail, also known as the Küntscher nail (K-nail) (Figure 1). The antibiotic-impregnated cement/polymer-coated intramedullary nail was prepared according to the manufacturer's instructions (Heraeus Medical, Hanau, Germany), and antibiotic selection was based on local antimicrobial susceptibility patterns. After insertion, the intramedullary nail was locked proximally and distally. The wound was then closed in layers, and a sterile dressing was applied.

Postoperative care

After analyzing the culture and sensitivity reports, the patient was administered intravenous antibiotics for a period of two to four weeks. The wound was inspected on the second or third day, and the dressing was done regularly if needed. Depending on the individual patient characteristics, the condition of the wound, and the specific organism involved, the patient would then be discharged and given oral antibiotics for an appropriate duration. Once the wound had healed, a patellar tendon-bearing cast or brace was applied, and the patient was permitted to gradually begin weight-bearing. Patients were instructed to begin weight-bearing as tolerated, with the use of crutches or a walker for support as needed. The cast was changed every six weeks and continued until clinical and radiological assessments confirmed the union. Active physiotherapy was initiated to regain ankle and knee mobility and continued until the range of motion was satisfactory. Patients were closely monitored in the weeks following their discharge and were seen for follow-up appointments once a week for the first month, then once a month for three months, and finally once every two to three months until the final follow-up appointment. Radiographs and laboratory data were collected at each follow-up visit. Any complications, including infection, implant failure, or nonunion, were recorded.

Outcome measures

The primary outcome measure was the proportion of patients with successful union of the fracture site, defined as the presence of radiographic evidence of bridging callus and no further need for surgical intervention. Secondary outcome measures included the time to union, the rate of deep infection, the rate of nonunion, and the need for revision surgery.

Statistical analysis

Statistical analysis was performed using IBM SPSS Statistics for Windows, Version 26.0 (Released 2019; IBM Corp, Armonk, New York, United States). Continuous variables were reported as median, range, means, and standard deviations, while categorical variables were reported as frequencies and percentages.

Ethical considerations

This study was conducted in accordance with the principles outlined in the Declaration of Helsinki and was approved by the institutional review board.

## Results

Table 1 presents the baseline characteristics of the 30 patients included in the study. Of the participants, 27 (90.0%) were male and three (10.0%) were female. The mean age of the patients was 39.76 years, with a standard deviation of 12.21 years, and the age range was between 23 and 67 years. In terms of fracture grade, 11 patients (36.7%) had closed fractures, while two (6.7%) had Gustilo-Anderson (GA) grade 1 fractures, three (10.0%) had GA grade 2 fractures, and 14 (46.7%) had GA grade 3 fractures (both A and B). Regarding the bone involved, 22 patients (73.3%) had tibia fractures and eight (26.7%) had femur fractures. The mean bone gap was 1.3 cm with a range of 1.0-1.9 cm, after initial debridement.

**Table 1:** Baseline characteristics of the patients (N=30). GA: Gustilo-Anderson.

Variables	N/mean±SD	%/Range
Gender		
Male	27	90.0
Female	3	10.0
Age (in years)	39.76±12.21	23-67
Fracture grade		
Closed	11	36.7
GA grade 1	2	6.7
GA grade 2	3	10.0
GA grade 3 (A and B)	14	46.7
Bone involved		
Tibia	22	73.3
Femur	8	26.7
Bone gap (cm)	1.3±0.3	1.0-1.9

Table 2 presents the outcomes of the procedure among the 30 patients. The median follow-up period was 8.21 months (range: 9.0-38.0). Among the patients, 28 (93.3%) had their infection controlled, while only two (6.7%) did not. Among the patients with infection control, 21 (70.0%) involved the tibia, while seven (23.3%) involved the femur. For bone union, 27 patients (90.0%) had successful bone union, while three patients (10.0%) did not. Among the patients with bone union, 20 (66.7%) involved the tibia, while seven (23.3%) involved the femur. Among the patients with successful bone union, 18 patients (60.0%) did not require an additional procedure, while nine patients (30.0%) did.

**Table 2:** Outcome of the procedure among patients (N=30).

Outcome	N/mean±SD	%/Range
Median follow-up period (months)	8.21±6.57	9.0-38.0
Infection controlled		
No	2	6.7
Yes	28	93.3
If yes, bone involved		
Tibia (n=22)	21	70.0
Femur (n=8)	7	23.3
Bone union		
No	3	10.0
Yes	27	90.0
If yes, whether additional procedure done		
No	18	60.0
Yes	9	30.0
If yes, type of additional procedure performed		
Bone grafting	5	16.7
Exchange interlocking with bone grafting	4	13.3
Rate of union (weeks)		
Tibia	22.13±6.39	(17-32)
Femur	17.21±5.45	(16-28)

**Figure 2:**
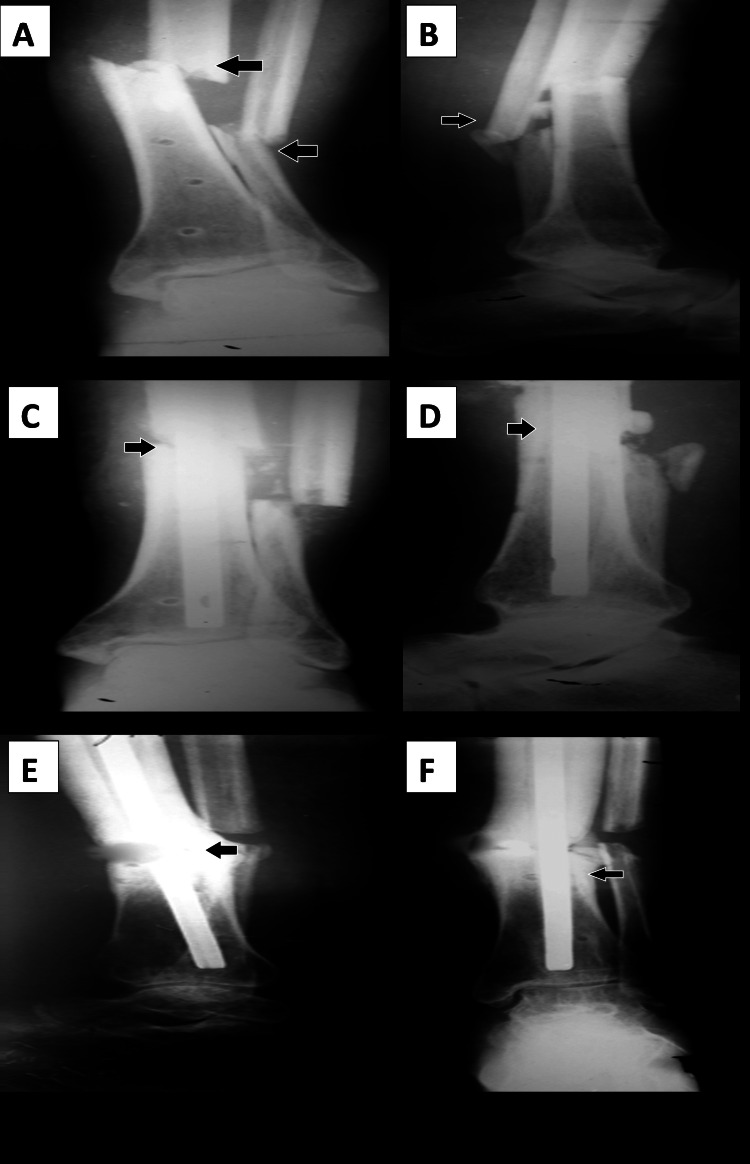
(A, B) Preoperative radiograph of a patient with infected nonunion of the tibia (arrows depicting nonunion tibia). (C, D) Postoperative radiograph (four weeks) of a patient with infected nonunion of the tibia (arrows depicting the alignment of nonunion tibia). (E, F) Postoperative radiograph (14 months) showing the bony union of a patient with infected nonunion of the tibia (arrows depicting the bony union of tibia).

Among the patients who required an additional procedure, bone grafting was performed in five patients (16.7%), and exchange interlocking with bone grafting was performed in four patients (13.3%). The mean rate of union was 22.13 weeks with a standard deviation of 6.39 weeks (range: 17-32 weeks) for patients with tibial fractures and 17.21 weeks with a standard deviation of 5.45 weeks (range: 16-28 weeks) for patients with femoral fractures (Figure 2).

Table 3 summarizes the outcomes of the procedure based on Paley's criteria among the 30 patients. For bony outcomes, 23 patients (76.7%) had excellent outcomes, two (6.7%) had good outcomes, two (6.7%) had fair outcomes, and three (10.0%) had poor outcomes. For functional outcomes, 21 patients (70.0%) had excellent outcomes, five (16.7%) had good outcomes, one (3.3%) had fair outcomes, and three (10.0%) had poor outcomes.

**Table 3:** Outcome of the procedure based on Paley’s criteria among patients (N=30).

Paley's criteria	N	%	N	%
	Bony outcome		Functional outcome	
Poor	3	10.0	3	10.0
Fair	2	6.7	1	3.3
Good	2	6.7	5	16.7
Excellent	23	76.7	21	70.0

Table 4 summarizes the complications observed among the 30 patients who underwent the procedure. The most common complications were persistence of bone nonunion, impingement of proximal nail, and debonding of nail cement, each occurring in three patients (10.0%). Persistence of infection was observed in two patients (6.7%), while nail breakage, nail bending, and migration of distal nails were each observed in one patient (3.3%).

**Table 4:** Complications of the procedure among patients (N=30).

Complications	N	%
Persistence of bone nonunion	3	10.0
Impingement of proximal nail	3	10.0
Debonding of nail cement	3	10.0
Persistence of infection	2	6.7
Nail breakage	1	3.3
Nail bending	1	3.3
Migration of distal nail	1	3.3

## Discussion

In this study, we evaluated the outcome of antibiotic-impregnated cement/polymer-coated intramedullary nails in the management of infected nonunion and open fractures of the femur and tibia. Our results demonstrated that this treatment modality led to a high rate of infection control and bone union, with excellent bony and functional outcomes based on Paley's criteria.

The baseline characteristics of the patients included in the study demonstrated a predominance of male patients with a mean age of 39.76 years. This is consistent with previous studies that have reported a higher incidence of these types of fractures in male patients and that these injuries tend to occur in younger patients [8,9]. In terms of fracture grade, 46.7% of the patients had GA grade 3 fractures, indicating a more severe type of injury. Additionally, 73.3% of the patients had tibia fractures, which are known to have a higher risk of complications and delayed healing compared to femur fractures [10,11].

Our study showed a high rate of infection control, with 93.3% of patients having their infection controlled. This is consistent with other studies by Yoon et al., Shyam et al., Qiang et al., Thonse et al., Paley et al., Dhanasekar et al., and Bhatia et al., which have reported similar success rates with antibiotic-impregnated intramedullary nails [12-18]. In a study by Paley et al., nine cases were evaluated, and all cases showed infection control [16]. Dhanasekar et al. reported successful infection control in 17 of 18 cases [17]. In another study by Bhatia et al., 19 of 20 patients (95%) showed successful infection control [18]. The use of antibiotic-impregnated cement/polymer coating on the nails can provide local delivery of antibiotics, which can help achieve high local antibiotic concentrations and reduce the risk of systemic side effects [19]. The high success rate of infection control in our study suggests that this treatment modality can be an effective option for managing infected nonunion and open fractures.

We also observed a high rate of bone union, with 90.0% of patients having successful bone union. This is consistent with previous studies by Yoon et al., Paley et al., Dhanasekar et al., and Bhatia et al., which have reported success rates ranging from 80% to 100% with antibiotic-impregnated intramedullary nails [12,16-18]. A study by Dhanasekar et al. reported that only three patients required exchange nailing to achieve bony union. In another study by Bhatia et al., bone union was observed in 18 out of 20 cases, with or without additional procedures [18]. The mean rate of union was 22.13 weeks for patients with tibial fractures and 17.21 weeks for patients with femoral fractures, which was shorter than the union rate mentioned in the study by Han et al. [20]. Although there was no control group in our study, these results suggest that antibiotic-impregnated intramedullary nails can lead to a relatively quick time to union.

Of the patients with successful bone union, 60.0% did not require an additional procedure. For those who required an additional procedure, bone grafting was performed in 16.7% of patients, and exchange interlocking with bone grafting was performed in 13.3% of patients. The need for additional procedures is consistent with previous studies by Dhanasekar et al., Bhatia et al., and Giannoudis and Tosounidis, which have reported similar rates of additional procedures with antibiotic-impregnated intramedullary nails [17,18,21]. Although additional procedures can increase the overall cost and length of treatment, the high success rate of infection control and bone union suggests that this treatment modality can still be a favorable option.

Finally, our study showed excellent bony and functional outcomes based on Paley's criteria. Specifically, 76.7% of patients had excellent bony outcomes, and 70.0% of patients had excellent functional outcomes. These results suggest that antibiotic-impregnated intramedullary nails can lead to favorable outcomes and may help patients return to their previous level of function.

Limitations

There are some limitations to our study that should be considered. Firstly, this was a single-center study with a relatively small sample size. Further studies with larger sample sizes and multi-center designs would be beneficial to confirm our results. Additionally, the lack of a control group limits our ability to draw conclusions about the efficacy of antibiotic-impregnated intramedullary nails compared to other treatment modalities.

## Conclusions

In conclusion, our study evaluated the efficacy and safety of antibiotic-impregnated cement/polymer-coated intramedullary nails in the management of infected nonunion and open fractures of the femur and tibia. The results demonstrated a high rate of infection control and bone union, with excellent bony and functional outcomes based on Paley's criteria. This treatment modality appears to be a valuable addition to the armamentarium of orthopedic treatments for infected nonunion and open fractures of long bones. The use of antibiotic-impregnated cement/polymer-coated intramedullary nails provides several advantages, including local antibiotic delivery, stabilization of the fracture, and early weight-bearing, which can lead to shorter rehabilitation periods. Moreover, it may reduce the need for prolonged antibiotic therapy, thereby potentially mitigating the risk of developing antibiotic resistance.
